# Bats adjust temporal parameters of echolocation pulses but not those of communication calls in response to traffic noise

**DOI:** 10.1111/1749-4877.12387

**Published:** 2019-10-22

**Authors:** Shengjing SONG, Aiqing LIN, Tinglei JIANG, Xin ZHAO, Walter METZNER, Jiang FENG

**Affiliations:** ^1^ Jilin Provincial Key Laboratory of Animal Resource Conservation and Utilization Northeast Normal University Changchun Jilin China; ^2^ Department of Integrative Biology and Physiology University of California Los Angeles California USA; ^3^ College of Life Science, Jilin Agricultural University Changchun Jilin China

**Keywords:** Chiroptera, chronic noise pollution, response latency, temporal parameters, vocal adjustment

## Abstract

Many studies based on acute short‐term noise exposure have demonstrated that animals can adjust their vocalizations in response to ambient noise. However, the effects of chronic noise over a relatively long time scale of multiple days remain largely unclear. Bats rely mainly on acoustic signals for perception of environmental and social communication. Nearly all previous studies on noise‐induced vocal adjustments have focused on echolocation pulse sounds. Relatively little is known regarding the effects of noise on social communication calls. Here, we examined the dynamic changes in the temporal parameters of echolocation and communication vocalizations of *Vespertilio sinensis* when exposed to traffic noise over multiple days. We found that the bats started to modify their echolocation vocalizations on the fourth day of noise exposure, with an increase of 42–91% in the total number of pulse sequences per day. Under noisy conditions, the number of pulses within a pulse sequence decreased by an average of 17.2%, resulting in a significantly slower number of pulses/sequence (*P* < 0.001). However, there was little change in the duration of a pulse sequence. These parameters were not significantly adjusted in most communication vocalizations under the noise condition (all *P* > 0.05), except that the duration decreased and the number of syllables/sequences increased in 1 type of communicative vocalization (*P* < 0.05). This study suggests that bats routinely adjust temporal parameters of echolocation but rarely of communication vocalizations in response to noise condition.

## INTRODUCTION

Anthropogenic noise disturbs acoustic communication, prey detection and predator avoidance in many animals (e.g. Brumm & Slabbekoorn 2005; Kight & Swaddle 2011; Siemers & Schaub 2011; Francis & Barber 2013; Senzaki *et al.* 2016; Shannon *et al.* 2016; Templeton *et al.* 2016), and animal response to noise is not only important from an ecological and biological perspective, but is also critical as an environmental factor with important consequences for conservation. Noise can negatively impact animal vocalization through acoustic masking, attention reduction and increased stress (Kight & Swaddle 2011; Francis & Barber 2013). Many studies based on acute short-term noise exposure have demonstrated that animals can adjust their vocalizations in response to ambient noise (e.g. Brumm 2004; Parris *et al.* 2009; Parks *et al.* 2011; Hage *et al.* 2013; Lowry *et al.* 2013; Duarte *et al.* 2018; Hill *et al.* 2018). To reduce the negative effect of noise, animals may adjust the spectral and/or temporal parameters of their vocalizations by increasing amplitude, frequency, bandwidth and duration of vocal elements (Parks *et al.* 2007; Rios-Chelen *et al.* 2012; Hage *et al.* 2013; Siegert *et al.* 2013; Hardman *et al.* 2017), and increasing the number of vocal elements and the repetition rate (Brumm *et al.* 2004; Roy *et al.* 2011; Luther & Gentry 2013; Caldart *et al.* 2016; Bittencourt *et al.* 2017; Nelson *et al.* 2017). However, the effects of chronic exposure to noise over a relatively long time scale of multiple days are less known (Barber *et al.* 2010; Cui *et al.* 2016; Kleist *et al.* 2018).

Bats are nocturnal flying mammals that rely mainly on acoustic signals for environmental perception and social communication. Echolocating bats not only produce echolocation signals for navigation, orientation and prey detection (Jones & Teeling 2006; Moss *et al.* 2011), but they also have rich vocal repertoires for individual recognition, mate attraction, predator avoidance and territorial defense (Kanwal *et al.* 1994; Bohn *et al.* 2008; Gadziola *et al.* 2012; Lin *et al.* 2016). Echolocation pulses are both functionally and acoustically distinct from communication calls. Echolocation pulses have a relatively simple and stereotypic spectrum, with most energy being concentrated at frequencies ranging from 20 to 150 kHz (Jones & Teeling 2006). In contrast, communication calls are often spectrally complex, with most energy being concentrated at frequencies below 25 kHz (Kanwal *et al.* 1994; Ma *et al.* 2006; Bohn *et al.* 2008; Gadziola *et al.* 2012; Lin *et al.* 2016).

Some species of bats inhabit or forage near manmade structures, such as bridges, buildings, mines and highways, which are noisy (Kunz & Fenton 2006). Among mammals, bats suffer the most from noise pollution. Noise can decrease the foraging activity and efficiency in bats (Schaub *et al.* 2008; Siemers & Schaub 2011; Bunkley & Barber 2015; Bunkley *et al.* 2015; Luo *et al.* 2015b). In response to noise, bats can adjust the acoustic characteristics of their echolocation pulses, such as amplitude, frequency and duration (e.g. Hage *et al.* 2013; Hage & Metzner 2013; Bunkley *et al.* 2015; Luo *et al.* 2015; Luo & Moss 2017). However, little is known about the impact of noise on social calls (but see Jiang *et al.* 2019). Moreover, the current knowledge on bat vocal adjustment to noise is based on short-term noise exposure, usually for only several minutes or less. It is unknown how bats might change their vocalizations, especially their temporal parameters, when exposed to chronic noise lasting several days or longer.

In this study, we examined how bats might change the temporal parameters of echolocation and communication vocalizations when exposed to chronic anthropogenic noise over multiple days. Temporal parameters were the focus of this study because the impact of noise on the spectral parameters of vocalizations has been largely described in bats (e.g.Tressler & Smotherman 2009; Hage & Metzner 2013; Hage 2013; Bunkley *et al.* 2015; Guo *et al.* 2015; Luo *et al.* 2015a,b; Jones *et al.* 2018). Asian particolored bats (*Vespertilio sinensis*) were collected from a colony that roosted beneath a traffic bridge and were exposed to the loud noises and vibrations associated with the passing vehicles. The bats in this bridge colony frequently emitted echolocation pulses and communication calls, with the dominant frequency of echolocation pulses at approximately 34 kHz and the majority of social calls ranging from 9 to 21 kHz (Fig. 1a,b). The frequencies of the traffic noise ranged from several hundred Hz to 70 kHz, with most energy concentrated at frequencies of 3–15 kHz (Fig. 1e).

## MATERIALS AND METHODS

### Location and animals

Twenty-six non-pregnant female adult *V. sinensis* were collected from a large colony inhabiting a traffic bridge in Harbin City, China. The bat colony roosts under the bridge from June to October each year and includes more than 10 000 individuals. The traffic volume on the bridge is high, with more than 500 vehicles per hour during the daytime (0830−1830 hours). Trucks represent more than 25% of the vehicles crossing the bridge.

The collected bats were housed at Northeast Normal University, Changchun, China. The bats were divided into 2 groups comprised of individuals with similar body size (forearm length: noise-exposure group 49.33 ± 1.39 mm; control group 50.45 ± 1.93 mm). The 2 groups were separately housed in cages (100 cm long

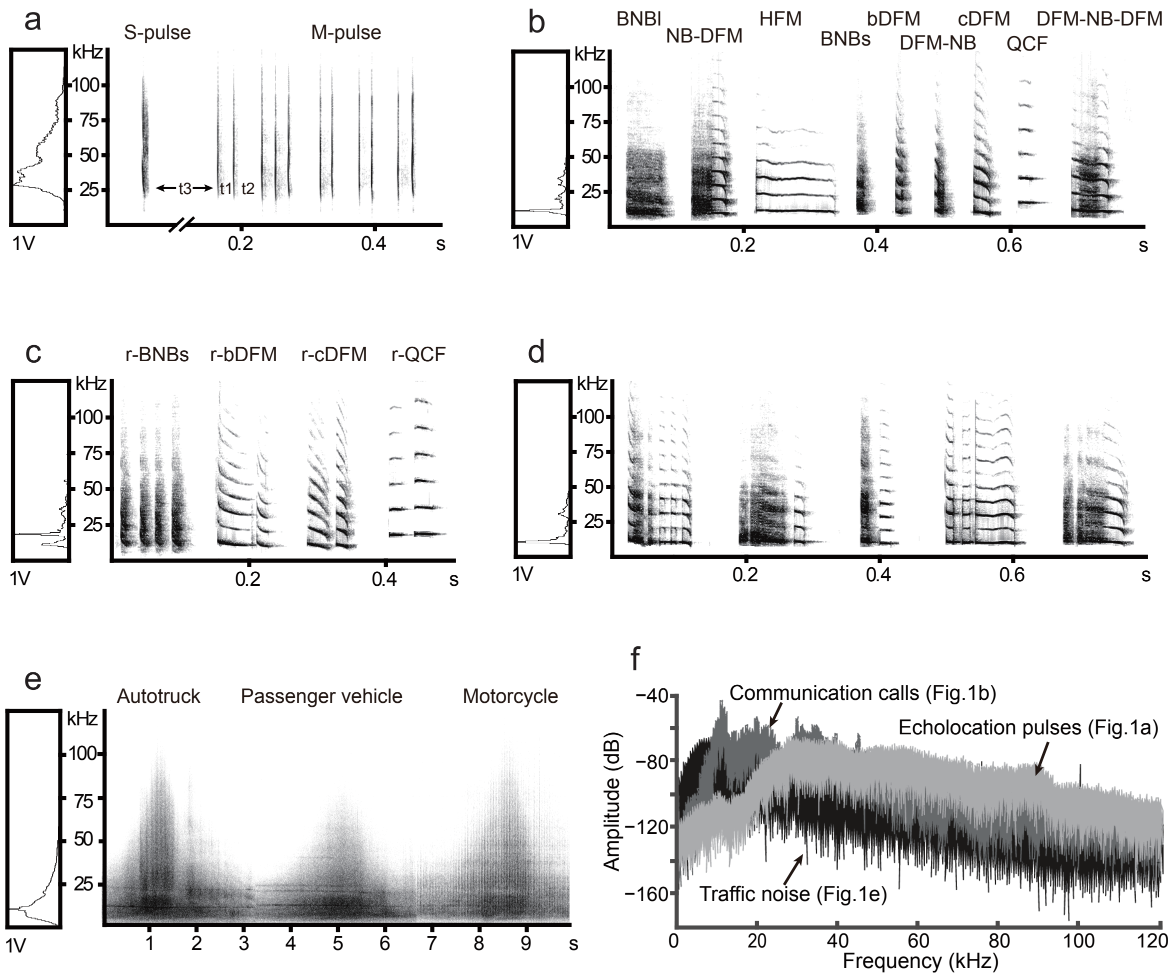

**Figure 1** Spectrograms of vocalizations of *Vespertilio sinensis* and traffic noise. (a) An example of echolocation single-pulse sequence (S-pulse) and multiple-pulse sequence (M-pulse). t1 and t2 refer, respectively, to the interval between pulses in doublet/triplet and the interval between doublet/triplet within a pulse sequence. t3 refers to the interval between 2 pulse sequences. (b) Nine distinct syllabic types of communication vocalizations. (c) The most frequently observed types of communication repeat-syllable call sequence. (d) An example of communication multisyllabic call sequences. (e) Traffic noise produced by the most common types of vehicles passing the bridge where the *V. sinensis* inhabit. The power spectrums (left) displayed in the figure from an average of all types. See Tables 1 and 2 for detailed abbreviations adopted for syllables and call sequences. (f) Power spectrum of echolocation (a) and communication (b) vocalizations and traffic noise (e) indicating the spectral overlap between summed energy in communication and echolocation vocalizations compared with that in playback noise. Noise had most energy present at frequency bands (3–15 kHz), which overlap with the part of bats' range of communication call frequencies (9–34 kHz).

× 60 cm wide × 80 cm high) in isolated rooms under the same conditions of temperature and illumination (temperature: 22−24 °C; relative humidity: 50−60%; light cycle 12-h light/12-h dark, 0700−1900 hours for light conditions; 4.80-m long × 2-m wide × 2.50-m high). They were given ad libitum access to water and mealworms, and their diet was enriched with vitamin and mineral supplements.

## Noise recording and generation of playback files

We recorded traffic noise on the bridge for acoustic playback. To obtain high-quality traffic noise, an ultrasonic microphone (UltraSoundGate CM16/CMPA, Avisoft Bioacoustics, Berlin, Germany) was placed at a distance of approximately 5 m from the nearest passing vehicles. The microphone was connected to an ultrasound recording interface (UltraSoundGate 116H, Avisoft Bioacoustics), with a sample rate of 375 kHz at 16 bits/sample. Traffic noise was recorded from 0830 to 1830 hours, with a total of 10 days of recording. The recorded files were automatically saved with a 1-min duration of each file. Each file was high-pass filtered at 1 kHz using Avisoft-SASLab Pro 5.2.

We measured the sound level of traffic noise in the location inhabited by the bats as a reference of amplitude for noise playback. A calibrated microphone (46BF, G.R.A.S. Sound & Vibration, Denmark) was positioned approximately 50 cm from the bats in the crevices under the bridge. The microphone was connected to an ultrasound recording interface (UltraSoundGate 116Hm, Avisoft Bioacoustics), with a sample rate of 375 kHz at 16 bits/sample. A 30-min recording (1000−1030 hours) showed that the traffic noise was approximately 63 dB SPL (sound pressure level re. 20 μPa). The noise bats experienced was louder than this level because the noise caused by vehicles can transmit via the bridge structure in addition to the air.

Fifty silence files, each 1-min long, were created using Avisoft-SASLab Pro 5.2, with a sample rate of 375 kHz at 16 bits/sample. These silence files did not have any acoustic signals and were broadcast to the bats of the control group.

## Acoustic playback and sound recording

In the first 14 days, the bats of both groups were maintained without any sound recording or playback. We then recorded the vocalizations of the bats to determine if there were any differences in the vocalizations between the 2 groups before playback. We recorded bat vocalizations from 0830 to 1830 hours each day and achieved a total of 5 days of recordings. Vocalizations were recorded using an ultrasonic microphone (UltraSoundGate CM16/CMPA) and interface (UltraSoundGate 116H), with a sample rate of 375 kHz at 16 bits per sample. Each microphone was placed at a distance of 1 m from the middle of the cage.

We then broadcast files of traffic noise to the bats of the noise-exposure group and silence files to the control group. Playback files were played by a loudspeaker (Ultrasonic Dynamic Speaker, Vifa, Avisoft Bioacoustics), which was positioned at a distance of 3 m from the cage. Each speaker was connected to an ultrasound playback interface (UltraSoundGate player 116, Avisoft Bioacoustics). Traffic noise was played with an amplitude of around 63 dB at the sites where the bats were hanging. A microphone (UltraSoundGate CM16/CMPA), connected to a recording interface (Avisoft UltraSoundGate 116H), was positioned at a distance of 1 m from the cage to record the vocalizations of the bats. The microphone and loudspeaker were parallel with each other and pointed to the same direction. Bat vocalizations were recorded with a sample rate of 375 kHz at 16 bits per sample. Traffic noise and silence files were respectively broadcast to the bats in the noise exposure group and the control group during 0830−1830 h each day for a total of 10 days of playback. The recorded noise files were sorted randomly by day and played to the bats in the noise-exposure group. The 50 silence files were randomly broadcast to the control group bats.

## Data analysis

An echolocation pulse sequence consists of either a single pulse (single-pulse sequence) or multiple pulses (multiple-pulse sequence). A communication call sequence consists of either a single syllable (single-syllable sequence) or multiple syllables (multisyllabic sequence). We measured the inter-pulse/syllable interval (duration between the end of a pulse/syllable and the onset of the next one) to establish the boundary of the pulse/call sequence. Two consecutive pulses/syllables were randomly selected from the database of vocalizations recorded and 1100 pulse/syllable intervals were measured respectively for echolocation and communication vocalizations of each bat group. *V. sinensis* often emit pulses in doublets and triplets (Fig. 1a), which results in a trimodal distribution of pulse intervals of echolocation vocalizations (Fig. S1a). The first trough was the boundary between 2 consecutive pulses within doublets and within triplets. We set the second trough as a boundary of pulse sequence so that 2 consecutive pulses were classified into 2 different pulse sequences when their interval was greater than 250 ms (Fig. S1a) or into the same pulse sequence if the interval was shorter than 250 ms. Similarly, the inter-syllable interval of 40 ms was set as a call sequence boundary for communication vocalizations (Fig. S1b).

A total of 92 296 sequences of echolocation and communication vocalizations were analyzed for the noise-exposure group and the control group. We counted the daily number of echolocation pulse sequences of each bat group: the number of single-pulse sequences, the number of multiple-pulse sequences and the total number of pulse sequences (including both single-pulse sequences and multiple-pulse sequences). In addition, we analyzed the following parameters: the duration of a single-pulse sequence, and the duration, number of pulses, and vocal rate within a multiple-pulse sequence. The vocal rate is the number of pulses divided by the duration of the pulse sequence. A daily mean value was calculated for each parameter.

*Vespertilio sinensis* produce multiple types of communication syllables (calls) (Fig. 1b–d). We classified syllable types following the methods reported in Kanwal *et al.* (1994). We then classified the communication call sequences into three categories: single-syllable call sequence (SS-call sequence: a call sequence consists of a single syllable frequently emitted in isolation), repeat-syllable call sequence (RS-call sequence: a call sequence consists of repeats of the same syllables) and multisyllabic call sequence (MS-call sequence: a multisyllabic call sequence). The numbers of communication call sequences per day in each bat group were analyzed as follows: the number of SS-call sequences, the number of RS-call sequences, the number of MS-call sequences and the total number of call sequences (including SS-call sequences, RS-call sequences and MS-call sequences). We analyzed the duration of each SS-call sequence, the duration and number of syllables, the vocal rate within each RS-call sequence and each MS-call sequence. A daily mean value was calculated for each parameter. For SS-call sequences and RS-call sequences, only the call sequences consisting of the most frequently observed syllables were selected for statistical analysis.

All the acoustic parameters were measured from spectrograms, with the Hamming window FFT = 512 and the overlap = 93.73%, using Avisoft-SASLab Pro v5.2. The normality of data was tested using the Kolmogorov–Smirnov test. The differences in daily mean values of each parameter between bats of noise-exposure and control groups were tested using an independent sample *t*-test or the Mann–Whitney *U*-test. All the statistical analyses were performed by SPSS 22 (IBM). To minimize bias, a blinded method was used so that the person who analyzed the acoustic data had no knowledge of the bat treatment.

## RESULTS

Vocal activities were not significantly different between bats of the noise exposure group and the control group during the first 5 days before playback. There was no significant difference in the daily total number of echolocation pulse sequences (noise exposure group, 1816 ± 42.61; control group, 1771.20 ± 256.73; independent samples *t*-test, $P > 0.05$; Fig. 2a), the daily number of single-pulse sequences (noise-exposure group: 577.20 ± 26.78; control group: 563.00 ± 4.00; independent samples *t*-test, $P > 0.05$; Fig. 2b) and the ratio of multiple-pulse sequences to the total number of echolocation pulse sequences per day (noise-exposure group: 68.24% ± 6.58%; control group: 67.81% ± 6.58%; independent samples *t*-test, $P > 0.05$). The daily total number of communication call sequences (noise exposure group: 1135.60 ± 417.09; control group: 1097.40 ± 233.47), the daily number of SS-call sequences (noise exposure group: 82.49% ± 3.58%; control group: 76.71% ± 6.28%), the daily number of RS-call sequences (noise exposure group: 5.04% ± 0.63%; control group: 4.88% ± 1.98%) and the ratio of the number of MS-call sequences to the total number of call sequences per day (noise exposure group: 12.47% ± 3.83%; control group: 14.40% ± 7.74%) were not significantly different between the 2 groups before playback (independent samples *t*-test, all $P > 0.05$).

### Numbers of vocalization sequences

The bats under noise exposure were more active in echolocation vocalization compared with those under silence playback. The average daily total number of echolocation pulse sequences of the noise-exposure group was 29.61% greater than that of the control group during the whole period of playback (noise exposure group: 2697.90 ± 702.89; control group: 1899.00 ± 15.00; independent samples *t*-test, $F = 0.91$, $P = 0.001$; Fig. 2a). On the first 3 days of playback, the differences in the total number of echolocation pulse sequences per day between the 2 groups of bats ranged from 0.80% to 12.31%. After 3 days, the differences increased and ranged from 42.19% to 91.25% (Fig. 2a). Bats exposed to noise emitted more single-pulse sequences but fewer multiple-pulse sequences per day, with an increased ratio of the number of single-pulse sequences to the total number of echolocation pulse sequences (independent samples *t*-test, $F = 2.85$, $P = 0.015$; Fig. 2b).

There was no significant difference in the communicative vocal activity between the 2 groups during the period of playback. The daily total number of communication call sequences (independent samples *t*-test, $F = 1.57$, $P = 0.464$; Fig. 2a) and the ratio of the number of call sequences of each call type to the total number of call sequences were not significantly different between the bats under noise exposure and silence conditions (independent samples $t$-test: SS-call sequence: $F = 4.28$, $P = 0.110$; RS-call sequence: $F = 3.89$, $P = 0.200$; MS-call sequence: Mann–Whitney $U$-test, $Z = 1.97$, $P = 0.052$; Fig. 2b). SS-call sequences accounted for approximately 80% of the daily total number of communication call sequences (noise exposure group: 82.96% ± 7.84%; control group: 75.61% ± 11.40%). The call sequences of s-QCF, s-bDFM and s-cDFM were the most frequently observed SS-call sequences, accounting for approximately 92% of the total number of SS-call sequences (Fig. 2c). The call sequences of r-BNBs, r-bDFM, r-QCF and r-cDFM accounted for approximately 78% of the number of RS-call sequences (Fig. 2d). These 7 most common call sequences as well as the MS-call sequences were selected for further analyses. The abbreviations for each syllable are shown in Tables 1 and 2.

## Duration of vocalization sequences

There was no significant difference in the duration of an echolocation single-pulse sequence (noise exposure group: 5.14 ± 1.15 ms; control group: 5.59 ± 0.73 ms; Mann–Whitney $U$-test, $Z = -1.74$, $P = 0.089$; Fig. 3a) or a multiple-pulse sequence (noise exposure group:

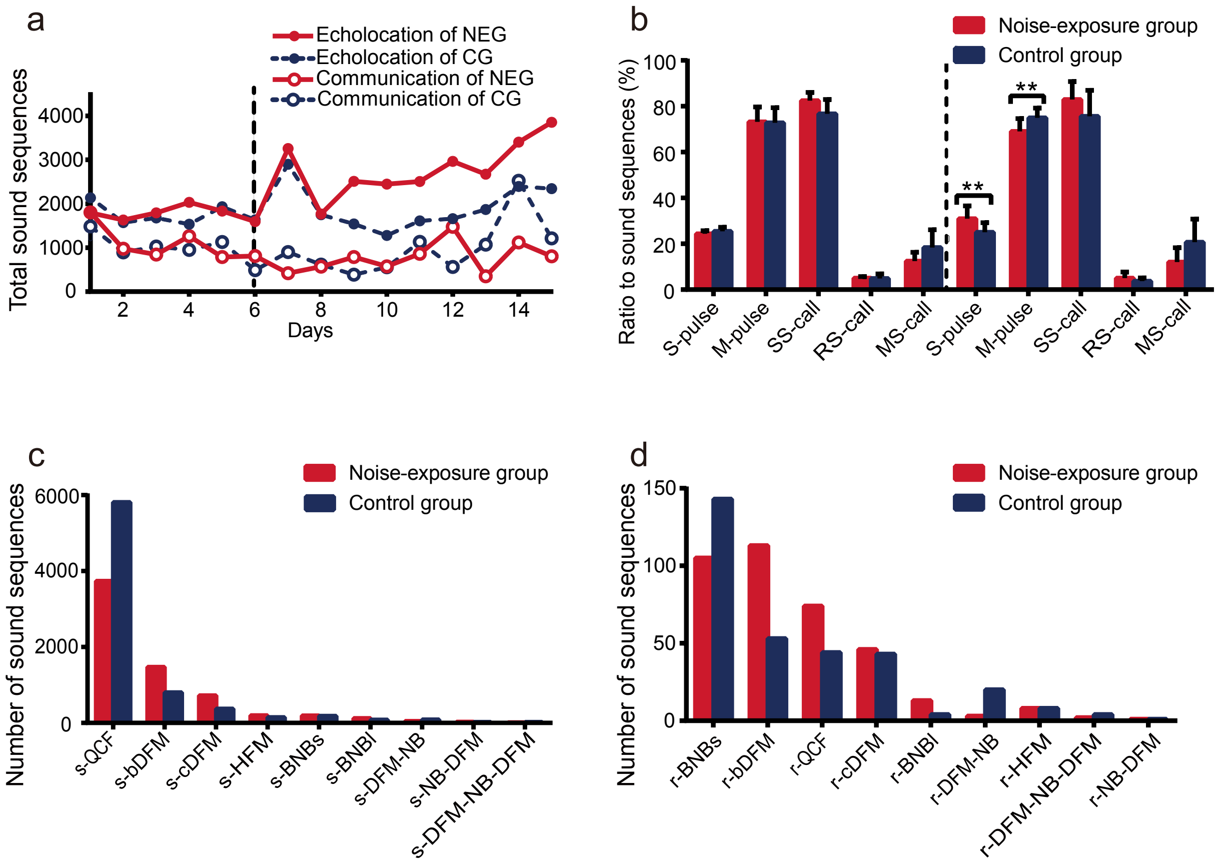

**Figure 2** Temporal parameters of echolocation and communication vocalizations in *Vespertilio sinensis* in the noise-exposure group (NEG) and the control group (CG) before and after playback. (a) The daily total number of sequences of echolocation (filled circle) and communication (open circle) vocalizations in bats in the noise-exposure group and the control group. (b) The ratio of the number of single-pulse sequences (S-pulse) or multiple-pulse sequences (M-pulse) to the number of total echolocation pulse sequences, and the ratio of the number of single-syllable call sequences (SS-call), repeat-syllable call sequences (RS-call) and multisyllabic call sequences (MS-call) to the total number of communication call sequences. The average daily total number of each type of communication SS-call sequences (c) and RS-call sequences (d) in the noise-exposure group and the control group after playback. Asterisks indicate a statistically significant difference (*$P < 0.05$, **$P < 0.01$). See Tables 1 and 2 for detailed abbreviations adopted for syllables and call sequences.

891.09 ± 96.43 ms; control group: 959.51 ± 121.15 ms; independent samples *t*-test, $F = 1.58$, $P = 0.197$; Fig. 3b) between the 2 bat groups. Under the noise condition, only the duration of r-cDFM call sequence was reduced significantly (noise exposure group: 111.88 ± 74.38 ms; control group: 219.77 ± 125.30 ms; independent samples *t*-test, $F = 1.94$, $P = 0.031$). The bats did not significantly change the duration of a call sequence of all of the remaining social communication call types when exposed to noise (all $P > 0.05$; Fig. 3a,b).

**Table 1** List of symbols and abbreviations of communication syllables of *Vespertilio sinensis*

| Symbols and abbreviations | Definition of abbreviations |
|---|---|
| BNBl | Broadband noise burst long syllable |
| HFM | Humped frequency modulation |
| BNBs | Short broadband noise burst |
| bDFM | Bend downward frequency modulation |
| cDFM | Checked downward frequency modulation |
| QCF | Quasi-constant frequency syllable |
| NB-DFM | Broadband noise burst-downward frequency modulation |
| DFM-NB-DFM | Two downward frequency modulations sandwiching a broadband noise |
| DFM-NB | Downward frequency modulation-broadband noise burst |

### Number of elements within vocalization sequences

The number of pulses within an echolocation multiple-pulse sequence was decreased by 17.2% in the bats of the noise-exposure group and was significantly less than the value of the bats in the control group (Mann-Whitney *U*-test, $Z = -2.87$, $P = 0.003$; Fig. 3c), resulting in a significantly slower vocal rate within an echolocation pulse sequence (noise exposure group: 10.88 ± 0.84; control group: 12.22 ± 0.48; independent samples *t*-test, $F = 0.56$, $P < 0.001$; Fig. 3d). Under noise conditions, the numbers of syllables and vocal rate within a call sequence did not change significantly in any communication call type (all $P > 0.05$; Fig. 3c), except for the call sequences of r-cDFM, within which the vocal rate significantly increased by 45% (Mann–Whitney *U*-test, $Z = -2.16$, $P = 0.031$; Fig. 3d). Although the duration and vocal rate was adjusted in r-cDFM, this vocal type accounted for only 1% of the total number of communication call sequences.

## DISCUSSION

Our results revealed that Asian particolored bats adjusted the temporal parameters of echolocation vocalizations but almost did not change those of communication vocalizations when exposed to chronic traffic noise. They increased the total number of echolocation pulse sequences, increased the number of single-pulse sequences, and decreased the number of pulses and vocal rate within multiple-pulse sequences. In contrast, all but one of the most frequently observed communication call

**Table 2** List of symbols and abbreviations of communication call types of *Vespertilio sinensis*

| Symbols and abbreviations | Define abbreviations | Symbols and abbreviations | Definition of abbreviations |
|---|---|---|---|
| s-BNBl | Single syllable of BNBl | r-BNBl | Repeat-syllables of BNBl |
| s-HFM | Single syllable of HFM | r-HFM | Repeat-syllables of HFM |
| s-BNBs | Single syllable of BNBs | r-BNBs | Repeat-syllables of BNBs |
| s-bDFM | Single syllable of bDFM | r-bDFM | Repeat-syllables of bDFM |
| s-cDFM | Single syllable of cDFM | r-cDFM | Repeat-syllables of cDFM |
| s-QCF | Single syllable of QCF | r-QCF | Repeat-syllables of QCF |
| s-NB-DFM | Single syllable of NB-DFM | r-NB-DFM | Repeat-syllables of NB-DFM |
| s-DFM-NB-DFM | Single syllable of DFM-NB-DFM | r-DFM-NB-DFM | Repeat-syllables of DFM-NB-DFM |
| s-DFM-NB | Single syllable of DFM-NB | r-DFM-NB | Repeat-syllables of DFM-NB |

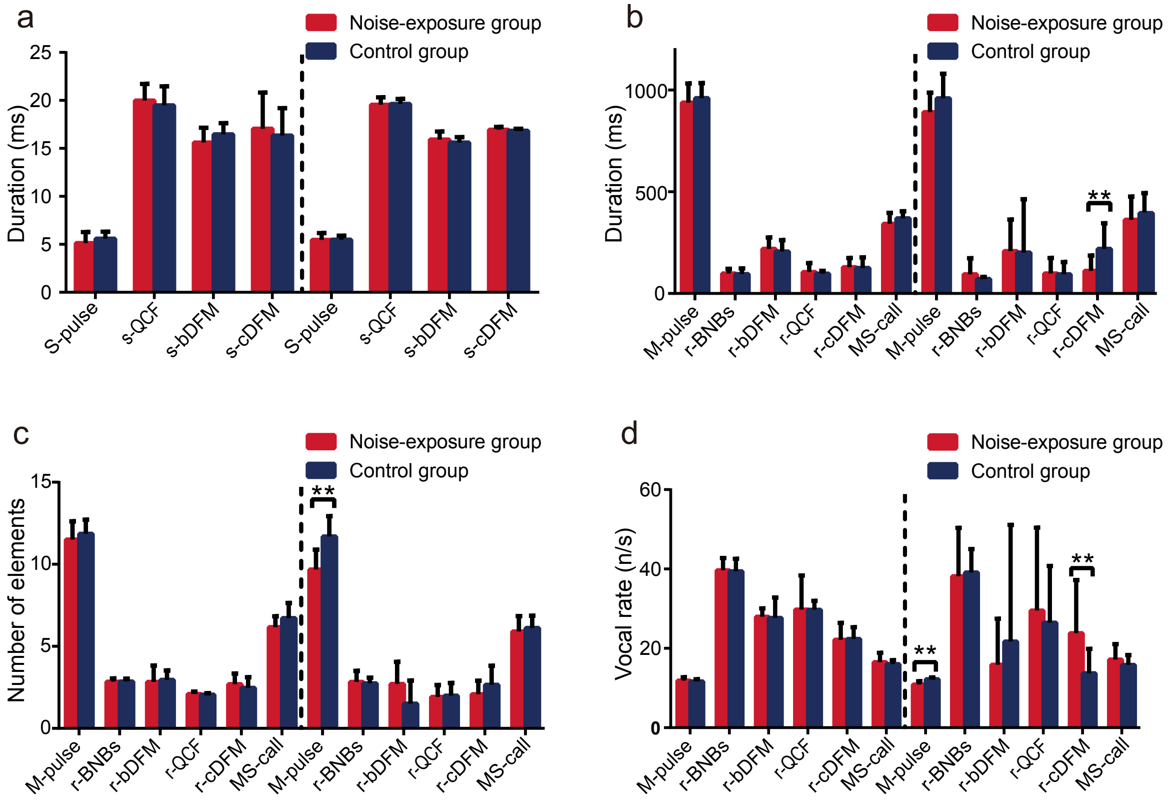

**Figure 3** Temporal parameters of echolocation and communication vocalizations in *Vespertilio sinensis* before and after playback: (a) Duration of echolocation single-pulse sequence (S-pulse) and duration of communication single-syllabic call sequence. (b) Duration of echolocation multiple-pulse sequence (M-pulse) and duration of communication repeat-syllable call sequence and multisyllabic call sequence (MS-call). The number of pulses/syllables (c) and the vocal rate (d) within an echolocation M-pulse, a communication repeat-syllable call sequence and an MS-call, respectively. The vertical dotted line indicates the results obtained before (left) and after (right) playback. Asterisks indicate a statistically significant difference (*$P < 0.05$, **$P < 0.01$). See Tables 1 and 2 for detailed abbreviations adopted for syllables and call sequences.

types were stable in these parameters. The total number of communication call sequences as well as the duration, number of syllables, and vocal rate within a communication call sequence were not significantly changed under the noise treatment.

## Increased echolocation activity under noise condition

The most obvious vocal response of the bats to traffic noise was an increased number of echolocation pulse sequences. One possible reason is that the vocal response increases the detectability of echolocation pulses. Anthropogenic noise can potentially mask high-frequency vocalizations because energy in the spectral region of a vocal signal also contributes to masking signals in other frequencies, albeit to a lesser extent (Kanwal *et al.* 1994; Parris & Schneider 2009; Díaz *et al.* 2011). Echolocation pulses of *V. sinensis* spectrally overlap with the high-frequency parts of the broadcast traffic noise, although the traffic noise has most of its energy in its low frequencies (Fig. 1f). Traffic noise may reduce the detectability of echolocation signals by acoustic masking. A recent study showed that traffic noise can affect the starting frequency, peak frequency and ending frequency of echolocation pulses in this species under natural conditions (Guo *et al.* 2015). The acoustic masking of echolocation pulses by anthropogenic noise in several other bat species such as *Antrozous pallidus* has also been suggested (Bunkley *et al.* 2015). An increase in the redundancy of acoustic signals can contribute to improving signal detectability and facilitating signal detection (Potash 1972; Wiley1994; Brumm & Slabbekoorn 2005; Luther & Gentry 2013; Luo *et al.* 2015a). Under noisy conditions, a signal sender repeating the same information may increase the probability of successfully transmitting the information during silent gaps between noise bursts (Roy *et al.* 2011; Velez & Bee 2011). Moreover, a receiver may extract an increasing amount of information from consecutive repetitions of the same signal, even in constant noise (Brumm & Slabbekoorn 2005; Brumm & Slater 2006). *V. sinensis* may increase the number of echolocation pulse sequences to improve the detectability of the signals in response to noise. This explanation is partly supported by the evidence that *V. sinensis* decrease call complexity to mitigate short-term (3 hours) noise interference during social communication (Jiang *et al.* 2019).

Adjustment of echolocation vocalization could also be a stressor response to the traffic noise. Noise exposure may act as a stressor to animals and thereby change physiological characteristics (Wright *et al.* 2007; Kight & Swaddle 2011), such as increasing stress hormone levels and inducing immunosuppressive effects that can alter vocal performance (Francis & Barber 2013; Grunst & Grunst 2014; Troïanowski *et al.* 2017). In this study, the bats exposed to noise did not significantly change their echolocation vocalizations until the fourth day of playback. This result differs from other bat studies testing short-term noise conditions where bats altered their vocalizations immediately when exposed to noise (e.g. Hage *et al.* 2013; Luo *et al.* 2017a). The different results may have been caused by the different acoustic parameters studied or they may reflect different underlying mechanisms. Improved signal detectability does not seem to be able to explain the 3-day latency of vocal adjustment in this study. Instead, the vocal-response latency is probably a result of accumulated noise stress and physiological responses. Under stress, behavioral response due to altered physiological processes may be relatively slow and delayed owing to the requirement of accumulating hormones, such as glucocorticoids (Romero & Butler 2007).

## Different responses of echolocation and communication vocalizations

The response of echolocation vocalization to noise has been reported in other bat species (Hage *et al.* 2013; Hage & Metzner 2013; Bunkley *et al.* 2015; Luo *et al.* 2017a,b). However, little is known about the impact of noise on bat communication vocalizations. The extent to which bats will respond to noise differently in echolocation and communication vocalizations is not known. Because masking is most effective when the masking sound spectrally overlaps with the target sound (Slabbekoorn 2013), it would be expected that noise masking of communication vocalizations would be stronger than on echolocation vocalizations and that the bats would alter communication calls more than echolocation pulses. Instead, our results showed that *V. sinensis*, in response to traffic noise, changed the temporal parameters of their echolocation pulses but barely changed those of communication syllables. Our finding is consistent with that of Jiang *et al.* (2019), who reported that *V. sinensis* did not increase syllable duration in response to short-term traffic noise.

One reason for the observed results in *V. sinensis* would be that the bats have a higher signal-noise ratio for the communication vocalizations than for the echolocation vocalizations and the bats have a sufficient signal-to-noise ratio with their communication calls to overcome the impact of traffic noise. Acoustic masking not only depends on the spectrum of the target signal and interfering noise, but also on the sound level of signal and noise as well as signal duration (Hotchkin & Parks 2013; Luo *et al.* 2018). Although our study did not control for the duration and amplitude difference between communication and echolocation vocalizations, it is obvious that the communication calls are much longer than the echolocation pulses and that the communication calls are high in amplitude. The absence of an observed change in the communication calls is probably due to the signal–noise ratio, with communication calls being sufficient to overcome the impact of noise. While the traffic noise has most energy concentrated at frequencies below the echolocation pulses, the traffic noise may not be strong enough to mask the echolocation pulses, but, importantly, strong enough to mask the echoes. The bats increase the vocal rate probably to stabilize the echo perception over repetitions of the echolocation pulses.

Another possible explanation is that the different response between echolocation and communication vocalizations was a result of a different response to physiological stress but not to acoustic masking. As mentioned above, the adjustments in echolocation vocalizations may be a stressor response to traffic noise. Animals possibly improve their vigilance in stressful conditions (Rozan *et al.* 2008). Bats use echolocation pulses mainly for environmental perception and use communication calls for social communication. Increasing echolocation pulse activity would promote the bats to obtain more environmental information. It is more likely that bats change their echolocation pulses than communication calls to increase vigilance. Bats integrate echolocation vocalization and vigilance within the limbic system of amygdala when overstimulated (Ma & Kanwal 2014). Traffic noise may trigger relatively specific areas of the limbic system for vigilance and eliciting increased echolocation pulse activity.

### Implications for conservation

The effect of anthropogenic noise on bat fitness remains unclear. Traffic and/or gas compressor station noise reduced the foraging activity and efficiency in *Antrozous pallidus* (Bunkley & Barber 2015), *Myotis daubentonii* (Luo *et al.* 2015b), *M. myotis* (Schaub *et al.* 2008; Siemers & Schaub 2011), *Tadarida brasiliensis* (Bunkley & Barber 2015), but not in *M. californicus*, *M. cillolabrum*, *M. lucifugus* or *Parastrellus hesperus* (Bunkley & Barber 2015). The impacts of noise appears to vary with species and the behavioral/physiological state of the animal (Luo *et al.* 2014). *V. sinensis* almost doubled the echolocation activity in traffic noise compared to the silence control. Echolocation is energetically costly for resting bats and increasing echolocation vocalization could result in higher energy costs (Speakman *et al.* 1989). We argue that even if noise exposure may not decrease fitness related to foraging, it may increase the energy expenditure due to more vocalization in bats. If the increased vocal activity reflects a stress response, the long-term stress could increase the incidence of disease (Romero & Butler 2007).

In conclusion, our study reveals that the Asian particolored bats change the temporal parameters of echolocation vocalizations but barely change those of communication vocalizations in response to chronic traffic noise. This suggests that vocal responses to anthropogenic noise can be inconsistent among different types of vocalizations and that the degree of spectral overlap between animal vocalizations and noise does not necessarily predict the level of response in temporal vocal parameters to chronic noise. This study has improved our understanding of how animals would adjust their vocalizations in response to chronic noise. Future work will advance our results by performing behavioral and physiological experiments to examine why bats increase their echolocation activity and how bats adjust the frequency and amplitude of their vocalizations when they are exposed to chronic noise.

## ACKNOWLEDGMENTS

We thank Heng Liu for bat collection and Bailu Si for help with data analysis. This work was supported by the National Natural Science Foundation of China (31500314 and 31872681 to A. L., 31670390 to J. F. and 31470457 to T. L.), the Fundamental Research Funds for the Central Universities (2412017FZ024 to A. L.), the Fund of Jilin Province Science and Technology Development Project (20180101024JC to T. L.) and the "1000 Talent Plan for High-Level Foreign Experts" from the Organization Department of the CPC Central Committee (WQ20142200259 to W.M.).

## SUPPLEMENTARY MATERIALS

**Cite this article as:**

## Cite this article as:

Song S, Lin A, Jiang T, Zhao X, Metzner W, Feng J (2019). Bats adjust temporal parameters of echolocation pulses but not those of communication calls in response to traffic noise. *Integrative Zoology* **14**, 576–88.

## Supporting information

Additional supporting information may be found in the online version of this article at the publisher's website.
**Figure S1** Frequency distribution of inter‐pulse/syllable intervals for echolocation pulse sequences (a) and communication call sequences (b) in *Vespertilio sinensis*. The arrow indicates the boundary of the pulse/call sequence.

Supporting Information
