# Supplementary material for: Bats adjust temporal parameters of echolocation pulses but not those of communication calls in response to traffic noise
Source: Integr Zool. 2019 Oct 22;14(6):576–88. doi: 10.1111/1749-4877.12387 (PMC6900015; doi:10.1111/1749-4877.12387)
Supplement: Supplementary file 1 — Supporting Information [file INZ2-14-576-s001.pdf]

Integrative Zoology 2019; 14: 576–588 doi: 10.1111/1749-4877.12387

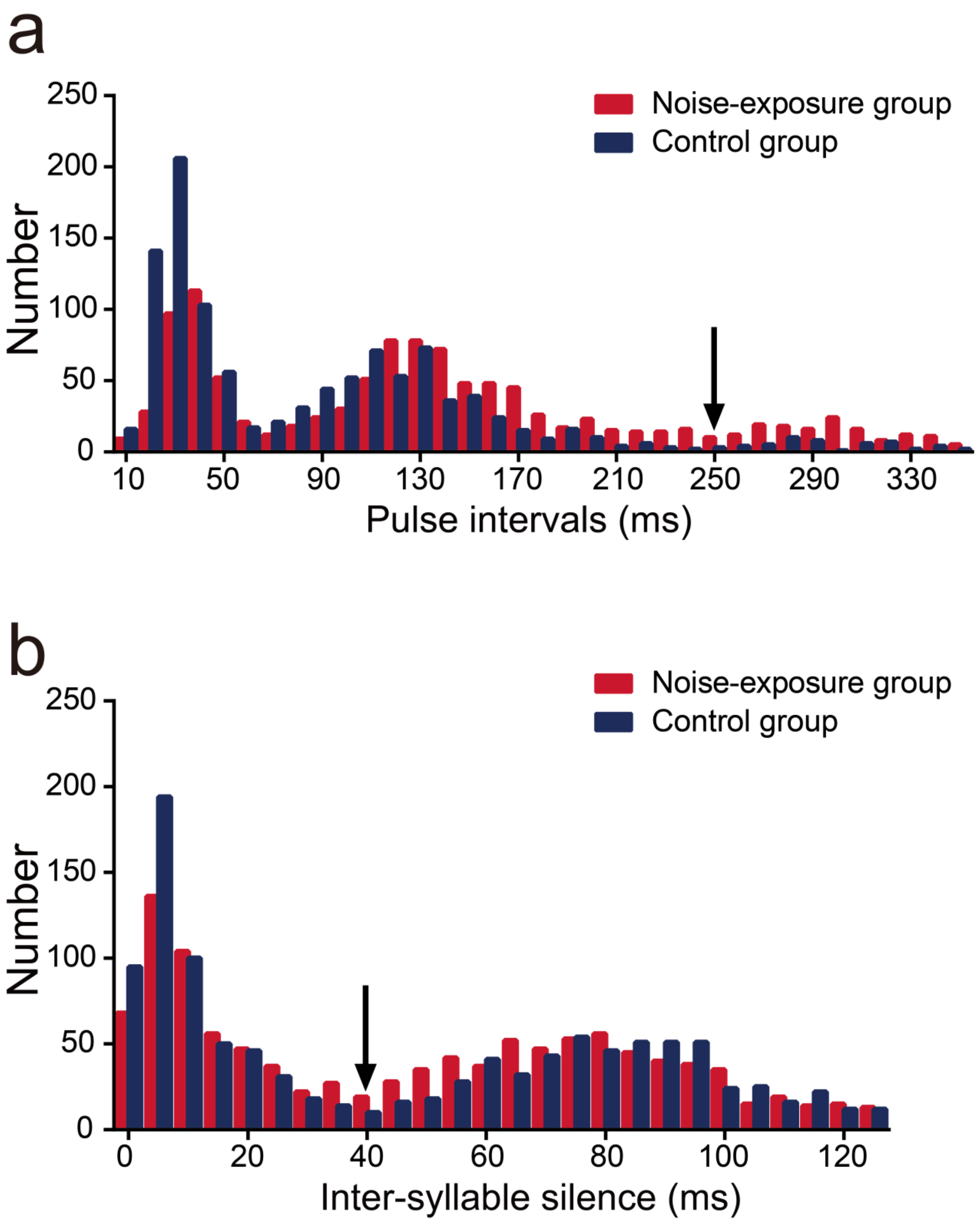

Figure S1 Frequency distribution of inter-pulse/syllable intervals for echolocation pulse sequences (a) and communication call sequences (b) in *Vespertilio sinensis*. The arrow indicates the boundary of the pulse/call sequence.
